# Topology driven modeling: the IS metaphor

**DOI:** 10.1007/s11047-014-9436-7

**Published:** 2014-06-24

**Authors:** Emanuela Merelli, Marco Pettini, Mario Rasetti

**Affiliations:** 1 School of Science and Technology, University of Camerino, Camerino, Italy; 2 Centre de Physique Théorique, UMR7332, Luminy, Aix-Marseille University, Marseille, France; 3 ISI Foundation, Turin, Italy

**Keywords:** Immune system, Multilinear mean field, Pattern discovery, Complex systems, Adaptive models, S[B] paradigm, Topology of data, Betti numbers, Big Data

## Abstract

In order to define a new method for analyzing the immune system within the realm of Big Data, we bear on the metaphor provided by an extension of Parisi’s model, based on a mean field approach. The novelty is the multilinearity of the couplings in the configurational variables. This peculiarity allows us to compare the partition function $Z$ with a particular functor of topological field theory—the generating function of the Betti numbers of the state manifold of the system—which contains the same global information of the system configurations and of the data set representing them. The comparison between the Betti numbers of the model and the real Betti numbers obtained from the topological analysis of phenomenological data, is expected to discover hidden *n-ary relations* among idiotypes and anti-idiotypes. The data topological analysis will select global features, reducible neither to a mere subgraph nor to a metric or vector space. How the immune system reacts, how it evolves, how it responds to stimuli is the result of an interaction that took place among many entities constrained in specific configurations which are relational. Within this metaphor, the proposed method turns out to be a global topological application of the S[B] paradigm for modeling complex systems.

## Introduction 

The objective pursued in this note is to frame the research on the immune system as part of *data science*. Such research is naturally complex and articulated and our contribution intends to be here along the lines of seeing it as a viable candidate for topological data analytics and an example of the S[B] paradigm for modeling complex systems. We recall that data science is the practice to deriving valuable insights from data by challenging all the issues related to the processing of very large data sets, while Big Data is jargon to indicate such a large collection of data (for example, exabytes) characterized by high-dimensionality, redundancy, and noise. The analysis of Big Data requires handling high-dimensional vectors capable of weaning out the unimportant, redundant coordinates. The notion of data space, its geometry and topology are the most natural tools to handle the unprecedentedly large, high-dimensional, complex sets of data (Carlsson 2009; Edelsbrunner and Harer 2010); basic ingredient of the new data-driven complexity science (TOPDRIM 2012; Merelli and Rasetti 2013).

Topology, the branch of mathematics dealing with qualitative geometric information such as connectivity, classification of loops and higher dimensional manifolds, studies properties of geometric objects (shapes) in a way which is less sensitive to metrics than geometric methods: it ignores the value of distance function and replaces it with the notion of connective nearness: *proximity*. All these features make topology ideal for analysing the space of data.

Starting from the notion of a mean field proposed by Parisi in his simple model for idiotypic network (Parisi 1990), we propose a more sophisticated version that is multilinear in the configurational variables (the antibody concentrations) instead of being constant or at most linear. Multi-linearity allows us to recognize in the partition function $Z$ of the model, that embodies all the statistical properties of the system at equilibrium, features similar to those of a particular *functor* of a topological field theory. The latter contains indeed the same global information about the topological properties (specifically its global invariants) of the system configuration space and can be identified with the generating function of Betti numbers, namely the Poincaré polynomial of data space (Atiyah and Bott 1983). Once the homology of the space of data has been constructed, and its generating cycles have been defined, the related two sets of Betti numbers can be compared. In this way, self-consistent information is obtained, regarding $2{\hbox {-}}ary$, $3{\hbox {-}}ary,\, \dots n{\hbox {-}}ary$ relations among antibodies. Comparison between the Betti numbers of the model and the real Betti numbers, obtained by constructing the topology of phenomenological immune system space of data, will unveil the hidden relations between idiotypes and anti-idiotypes; in particular, those relations where components interact indistinctly and therefore can not be reduced to a mere subgraph, but rather they bear on a new concept of interaction, scale-free and metric-free. The analysis of Betti numbers on phenomenological data can be dealt with techniques based on persistent homology (Carlsson 2009; Petri et al. 2013).

The challenge we are facing is to unveil whether in natural, multi-level complex systems, $n$-body interactions can drive the emergence of novel *qualia* in these systems. In physics, the interactions between material objects in real space are binary. This means that mutual forces and motions are produced by two-body interactions, the building blocks of any many-particle system. Thus at the atomic or molecular level description of matter (living or not) the total force acting on any given particle is the result of the composition of binary interactions. However, how can we discover if $n$-body interactions do exist? What we are proposing here is to use the IS metaphor, i.e. a complex system whose adaptivity is driven by data, as a global topological application of the S[B] paradigm. S[B] allows us to entangle in a unique model the computational component with the coordination. In particular, B accounts for the computation while S describes the global computation context (Merelli et al. 2013). The adaptation phase occurs when a machine can no longer compute in a given state of the system, thus the system changes state, i.e. the global context of computation. In the IS metaphor the computation context can be identified by the global invariants while the computation with the model of interactions, a sort of interactive machine. Each time we discover new global invariants, a new context of computation arises and with it a new IS model must be generated; we call this step the adaptation phase.

In the following, after giving a brief description of the antigen-free immune system and recalling Parisi’s mean field model, we formally define the new topological field model, and, finally, discuss the S[B] paradigm. An appendix is provided with a general introduction to the fundamental tool of *persistent homology and Betti numbers*.

## The antigen-free immune system

**Fig. 1:**
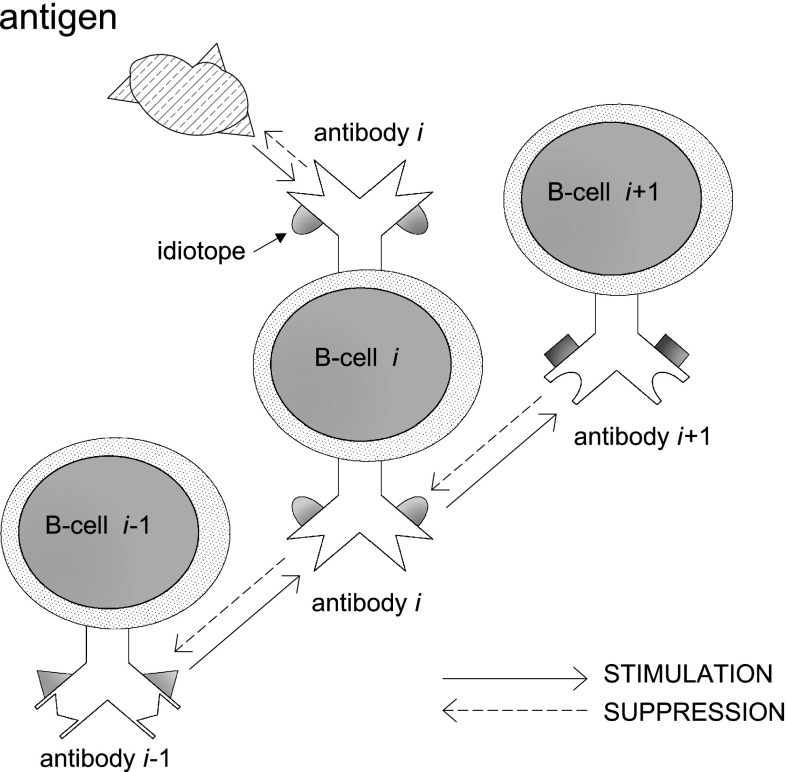
Jerne’s idiotypic network; $2$-body interactions

Cells and molecules of the immune system not only recognize foreign substances; they react and regulate each other, so that the immune system can be seen as a network of interacting cells and antibodies. This perspective is known as the idiotypic or immune network theory (Jerne 1974). It refers to the immune system as a complex process that takes place at the cellular level for protecting organisms from infectious agents (the *antigens*), which are antibody generators. In the scheme proposed by Jerne, it is the antigen that provokes an immune response and each antibody is represented as a large Y-shaped protein. The immune system uses this protein to identify and neutralize foreign objects. The antibody can recognize and bind a specific part of the antigen; resorting to this binding mechanism it can block the attack. Moreover, in Jerne’s network theory, antibodies are capable of being recognized by other antibodies; whenever this happens the former is suppressed and its concentration is reduced while the latter is stimulated and its concentration increases (see Fig. 1).

The mechanism whereby the production of a given antibody elicits or suppresses the production of other antibodies that, in turn, elicit or suppress the production of other antibodies like a concatenation of events, hints to a strict analogy of the immune system function with memory in the brain. It recalls the way in which a firing neuron may induce or inhibit the firing of other neurons, and so forth. On the assumption that a functional network of antibodies is possible, several models have been constructed, among which Parisi’s model. The latter studies the persistence of immune memory in the absence of any driving effect of external antigens and it offers a robust, though simple, theoretical framework without providing detailed description of the system (Parisi 1990).

The model we propose is a preliminary test of data field theory; it aims at a deeper understanding of the functional properties that the global and persistent topological properties of an antibodies data space can imply. In particular, it targets at discovering the existence of $n$-ary relations among antibodies and determining how the ensuing configurations influence the immune system reaction to the presence of antigens. The extraction of global qualitative information from an antibodies data space (e.g. concentrations), should lead to the discovery of those characteristics that are shared in a group of immunoglobulin receptor molecules. This means not only discovering a single idiotype, but the capacity of being active in the presence of $n$ others. We want to prove that topological data analysis, through persistent homology and its Betti numbers, allows us to determine the effective $n$-antibody configurations. Note that the models proposed in literature to describe the relationship between structure and function in biological networks are all based on the concept that any relation can be reduced to a set of binary relation (Hart et al. 2009): we argue that this is not necessarily the case. We start thinking of models as relationships, i.e. facts in logical space of forms. Forms that can be directly classified by Betti numbers, extracted by calculating the Betti numbers through the persistent homology of the space of data and used in the frame of a conceptual model able to bear on those topological features.

### Parisi mean field model for IS

The simplest and most efficient network of the immune system is represented by a model that can be easily formulated in the absence of antigens. Although it is well known that the number of specific lymphocytes plays a crucial role, the variables of the network model are limited to the antibody concentrations.

The mean-field idiotypic network model of antigen-free Immune System, proposed by G. Parisi and inspired by an earlier Hopfield’s model conceived to represent the brain and many other similar models (Hopfield 1982; Hoffmann 1975, 2010; Farmer et al. 1986; Varela et al. 1988), describes essentially an iterated cascade of events, in which the production of a given antibody provokes, or possibly inhibits, the production of other antibodies, which in turn induce, or possibly impede, the production of other antibodies, which in turn give rise to or prevent the production of other antibodies, etc..

In the Parisi model, the concentration $c_i(t)$ of antibody $i$ is assumed to have, in absence of external antigens, only two values, conventionally $0$ or $1$ (in the presence of antigen concentrations $c_i$ might become $\gg 1$); $t$ is time. The immune system state at time $t$ is determined by the values of all $c_i$’s for all possible antibodies $(i = 1, \dots , N)$. The dynamical process is typically described by a discretized time (the time step $\tau$ being the time needed to implement the immune response). The dynamical variable $h_i$ (the *mean field*) represents the total stimulatory/inhibitory (depending on its sign) effect of the whole network on the $i$-th antibody. $h_i$ is positive when the excitatory effect of the other antibodies is greater than the suppressive effect and then $c_i$ is one. Otherwise $h_i$ is negative and $c_i$ is zero. The mean-field is expressed typically as

1 $$\begin{aligned} \displaystyle {h_i (t)= S + {{\mathop {\mathop {\sum }\limits _{k=1}}\limits _{k \ne i}^{N}}} J_{ik} c_k (t)}, \,\,\, where \,\,\, c_i (t) = \Theta [h_i (t-\tau )] \end{aligned}$$

$\Theta (x)$ denotes the Heaviside function that is zero for negative $x$ and 1 for positive $x$, while $J_{ik}$ ($J_{ii} = 0, J_{ki} = J_{ik}$) represents the influence of antibody $k$ on antibody $i$. If $J_{ik}$ is positive, antibody $k$ triggers the production of antibody $i$, whereas if $J_{ik}$ is negative, antibody $k$ suppresses the production of antibody $i$. $\left| J_{ik} \right|$ is a measure of control efficiency that the antibody $k$ exercises on antibody $i$. The $J_{ik}$ are distributed in the interval $[-1, +1]$. $S$ is the threshold parameter; it regulates the dynamics when the couplings $J_{ik}$ are all very small; otherwise $S$ is equal to zero. At equilibrium, when the concentrations of antibodies are time independent, the Eq. (1) simplifies to

2 $$\begin{aligned} \displaystyle {h_i=S+ {{\mathop {\mathop {\sum }\limits _{k=1}}\limits _{k \ne i}^{N}}} J_{ik} c_k}\, , \, c_i= \Theta (h_i )\in \{ 0 , 1 \} \; . \end{aligned}$$

This idiotypic network model has the advantage of being simple and easy to analyze. The phenomenon of dependence of the immunity/tolerance pathway on the amount of antigens suggests that the concentration of any given antibody is crucial to determine the effects on the other antibodies. The assumption of two levels of concentration (0 or 1) bypasses the problem of the choice of a pathway.

However, this model is elementary in view of testing the perspectives of a data field theory. We need to increase its complexity in order to reach a description of the system sufficiently detailed to catch the global features of its data space. We generalize the mean field in such a way that it crucially depends on those topological features of the space of antibody concentrations that will be reflected in the topological properties of the system space of data. We construct a model sensitive to global features, designed to benefit of the advantage of lending itself to a kind of *reverse engineering* of the process of field construction. In the model, the antibodies with positive $c_i \, (=1)$ are actually produced by the system while those absent $(c_i=0)$ are suppressed. Suppression due to clonal abortion is neglected.

## The topological field model for antigen-free immune system

In this section, we generalize the way how Parisi’s linear model represents immunological memory by a linear mean field. The antibodies of the idiotypic cascade are denoted by $Ab_i$; during the production of $Ab_1$, ignited directly by the antigen, the environment of lymphocytes is modified by $Ab_2$: the life-span of the $Ab_1$-producing cells and the population of helper cells specific for $Ab_1$ increase. The symmetry of the couplings $(J_{ik}=J_{ki})$ implies that $Ab_3$ should be rather similar to $Ab_1$, the internal image of $Ab_2$ should persist after it disappeared, its presence induces the survival of memory cells directed against the antigen. The process continues by iteration. In the extended model, we assume the production of $Ab_i$ is conditioned to different extents and also by the simultaneous presence of a subset of $2, 3, ... , N$, antibodies.

A weakness of this representation is that the possible equilibrium configurations of the network are fixed, whereas we want the network to be capable of learning which antibodies should be produced without assuming that only a fraction of all antibodies have physiological relevance. Therefore, whilst we maintain the global cost function

3 $$\begin{aligned} \displaystyle { E= \sum _{i=1}^N h_i c_i }, \, \,\, \; c_i= \Theta (h_i )\in [0,1] \; , \end{aligned}$$

we consider in the space of antibodies $\mathcal {A}$, the points of which are labelled by $i=1 \dots ,N$, the graph $\mathcal {G}$ generated by the $J_{ik} \ne 0$ (for simplicity we assume here that $J_{ik}\in [ -1, +1 ]$ when $J_{ik} \ne 0$). We next extend $\mathcal {G}$ to the simplicial complex $\mathcal {C}$, obtained from $\mathcal {G}$ by completion, constructing the simplicial complex $\mathcal {C}$ which has $\mathcal {G}$ as 1-skeleton (scaffold), see Fig. 5. Each $n$-cycle in $\mathcal {C}$ cannot be seen as composition of two-body interactions, but represents a true $n$-body interaction; in other words, any relationship expressed in the cycle is unique in its configuration. We denote by $C^{(n)} ([l_1, \dots l_{(n+1)} ]$) the cycles of $\mathcal {C}$, and by $\delta _{k,i}$ the presence or the absence of $i$ in the cycle ($\delta _{k,i}=1$ if $k=i$, $\delta _{k,i}=0$ if $k\ne i$) and we generalize then the standard linear form for the mean field $h_i$ to the form:

4 $$\begin{aligned} h_i=S+ \sum _{k = 1}^N \, {\mathop {\mathop {\sum }\limits _{C^{(n)} ([ \ell _1, \dots \ell _{(n+1)} ])}}\limits _{1 \le n \le N - 1}} J_{\ell _1 \dots \ell _k \dots \ell _{n+1}} \prod _{j=1}^n c_{\ell _j} \, \delta _{k, i} \; \end{aligned}$$

In the partition function

5 $$\begin{aligned} \displaystyle {Z(x) \doteq \sum _{\left\{ c_\ell \right\} } e^{-x E\left( \left\{ c_\ell \right\} \right) }} \; \; x \in \mathbb {R}, \end{aligned}$$

the sum runs over the set of all possible valuations $c_\ell = 0 , 1 \; , \; \forall \ell$, subdivides the set of states in classes of equivalence, giving different statistical weights—depending on a parameter $x \in {\mathbb {R}}\; , \; x >0$—to those states which are invariant with respect to a given set of transformations. A phase transition, if any, would allow us to pass from one class of equivalence to the other when the state symmetry is (partially or fully) broken. This turns the model into a theoretical framework where, given a parameter—for example the average specific antibody concentration—we can predict when and if a configuration may break into another, giving rise to a different immunity type, i.e. change the adaptive immunity. In terms of formal language theory, going from one configuration to another belonging to a different class of equivalence has the following meaning: if we associate to the space of data a group of possible transformations preserving its topology (e.g., its mapping class group), and the related regular language, the general semantics thus naturally generated describes the set of all transformations and hence of all ‘phases’ in the form of relations.

We consider then the functor partition function, $Z(x)$. We might of course access more information (patterns) by considering higher ($k$-th) order correlation functions,

6 $$\begin{aligned} \Gamma _k (x) \doteq \frac{1}{Z(x)} \sum _{\left\{ c_\ell \right\} } c_{\ell _1} \dots c_{\ell _k} e^{-x E\left( \left\{ c_\ell \right\} \right) } \; , \end{aligned}$$

for any given set of points ${\ell _1 \dots \ell _k } \in \mathcal {A}$. We can represent with strings of $N$ dichotomic variables the set of $\{c_\ell \}$, $2^{N-1}$ possible configurations.

A crucial assumption we add to the model is that the coupling constants $J_{\ell _1 \dots \ell _k \dots \ell _{n+1}}$ are taken to be proportional to a linear combination (with negative coefficients) of the simplex $n$-volume $V^{(n)}$, the simplex corresponding to the cell defined by the set $\bigl \{ \ell _1 , \dots , \ell _n \bigr \}$ in the cells of cycle $C^{(n)} ([\ell _1, \dots , \ell _{(n+1)} ])$, with the volume of the cell boundary of dimension $n-2$, weighted by the curvature at that boundary. The latter measures the ease with which the $n$-body interaction is favored by the manifold bending. The ensuing action is expected to measure reasonably well the probability that the $n$-body process described by that coupling takes place.

When the model with such interaction form is dealt with as a statistical field theory it turns out to be fully isomorphic with a Euclidean topological field theory describing a totally different physical system: gravity coupled with matter in a simplicial complex setting, consistent with general relativity. We think back to the standard example of the Ising model, which also has variables in ${\mathbb {Z}}_2$ (Parisi 1998) and recall that a statistical field theory is any model in statistical mechanics where the degrees of freedom comprise a field; i.e. the microstates of the system are expressed through field configurations. The features of the ensuing theory are quite general and far reaching. The topology of the associated moduli space depends only on the manifold genus $g$, on the dimension $n$ of the (vector) bundle over it used to define the field, and on the dimension $\delta ({\mathrm{mod}} \, n)$ of the associated determinant bundle. Such space is a projective variety, smooth only if $(\delta ,n) = 1$. The recursive determination of the Betti numbers in this case is given by the Harder and Narasimhan and Atiyah and Bott recursions (Harder and Narasimhan 1975; Atiyah and Bott 1983). The former explicitly counts points of the moduli space, the latter resorts to an infinite-dimensional Morse theory with the field action functional as Morse function. These recursions lead to a closed formula for the Poincaré polynomial, i.e. for the Betti numbers of the moduli space. These implicit methods were successively made explicit (Desale and Ramanan 1975).

What is intriguing is that our field theory turns out to be isomorphic to ${\mathbb {Z}}_2$ (quantum) gravity, dealt with in nonperturbative fashion by standard Regge calculus (Regge 1961).

Let us recall here that the construction of a consistent theory of quantum gravity in the continuum is a problem in theoretical physics that has so far defied all attempts of a rigorous formulation and resolution. The only effective approach to try and obtain a non-trivial quantum theory proceeded via discretization of space-time and of the Einstein action, i.e., by replacing the space-time continuum by a combinatorial simplicial complex and deriving the action from simple physical principles.

Quantum Regge calculus, based on the well-explored classical discretization of the Einstein action due to Regge, and the essentially equivalent method of dynamical triangulations are the tools that proved most successful. Regge’s method consists in approximating Einstein’s continuum theory by a simplicial discretization of the space-time (in gravity a four-dimensional Lorentz manifold) resorting to local building blocks (simplices) and then constructing the gravitational action as the sum of a term depending on the (hyper)volumes of the different simplicial complexes and another reflecting the space-time curvature. The metric tensor associated with each simplex is expressed as a function of the squared edge lengths, which are the dynamical variables of this model. Summing over all interpolating geometries (state sum) generated by the simplicial complex construction in the embedding higher-dimensional ones (filtration), allows us to derive both the Einstein action and the equilibrium configurations simply by means of counting procedure (entropy estimate).

The ${\mathbb {Z}}_2$ version of the model is one in which representations of $SU(2)$ labeling the edges in quantum Regge calculus are reduced to ${\mathbb {Z}}_2$. The power of the method resides in the property that the infinite degrees of freedom of Riemannian manifolds are reduced by discretization; and the theory can deal with PL spaces, described by a finite number of parameters. Moreover, for the manifolds approximated by a simplicial complex (or by dynamically triangulated random surfaces), the local coordination numbers are automatically included among the dynamical variables, leaving the quadratic link lengths $q_\ell$, globally constrained by triangle inequalities, as true degrees of freedom.

More precisely, the model adopted here for the immune system is isomorphic to the ${\mathbb {Z}}_2$ Regge model, where the quadratic link lengths $q_\ell$ of the simplicial complexes are restricted to take on only two values: $q_\ell = 1 + {\mathfrak {l}} \sigma _\ell$, where $\sigma _\ell = \pm 1 = 2 c_{\ell } - 1$. Such model has been exactly solved (in the case of quantum gravity) via the matrix model approach (Ambjørn et al. 1985) and with the help of conformal field theory (Knizhnik et al. 1988). A crucial ingredient is the choice of functional integration measure, whose behavior, with respect to diffeomorphisms, is fundamental. The very definition of diffeomorphism is a heavy constraint in constructing the PL space exactly invariant under the action of the full diffeomorphism group (Menotti 1998), and only the recent construction of a simplicial version of the mapping class group made it viable (Merelli and Rasetti 2013).

As Regge regularization leads to the usual Liouville field theory in the continuum limit based on a description of PL manifolds with deficit angles, not edge lengths, we may assume that also in our case the correct measure has to be nonlocal. Starting point for the ${\mathbb {Z}}_2$ Regge model is a discrete description of general relativity in which space-time is represented by a piecewise flat, simplicial manifold (Regge skeleton). The procedure works for any space-time dimension $d$, metrics of arbitrary signature, and action

7 $$\begin{aligned} A ( \mathbf{{q}} ) = x \left( \sum _{s^d} V^{(d)} \left( s^d \right) - \zeta \sum _{s^{d-2}} {\mathfrak {d}} ( s^{d-2} ) \, V^{(d-2)} \left( s^{d-2} \right) \right) \; \end{aligned}$$

with the quadratic edge lengths $\left\{ q_\ell \right\}$ (more precisely, the $\sigma _{\ell }$’s) describing the dynamics of the complex. $x$ and $\zeta$ denote free constants (in the discrete time picture, with uniform time step $\tau$, energy functional and action are merely proportional). The first sum runs over all $d$-simplices $s^d$ of the simplicial complex, while $V ( s^d )$ is the $d$-volume of $s^d$. The second term represents the curvature of the simplicial complex, concentrated along the $(d - 2)$-simplices, leading to deficit angles ${\mathfrak {d}} ( s^{d-2} )$. The physical meaning of the terms entering action $A$ is what makes it acceptable for a consistent description of the immune system with higher order (‘many body’) interactions: the lower the volumes and the higher the curvature, the lower is the action $(x, \zeta > 0)$.

At equilibrium, i.e. in the absence of an explicit time-dependence of the expectation values of the variables, the partition function for our antigen-free IS model is nothing but the field propagator of the theory, expressed via path integral

8 $$\begin{aligned} \displaystyle {Z = \int \mathcal{{D}} \, [\mathbf{{q}}] \, \mathrm{{e}}^{- A ( \mathbf{{q}} )}} \end{aligned}$$

Functional integration should extend over all metrics on all possible topologies, hence the path-integral approach, typically suffers from a nonuniqueness of the integration measure and a need for a nonlocal measure is advocated. The standard ‘simplicial’ measure

9 $$\begin{aligned} \displaystyle { \int \mathcal{{D}} \, [\mathbf{{q}}] = \prod _\ell \, \int \frac{\mathrm{{d}} q_\ell }{q_\ell ^\alpha } \, \mathcal{{F}} ( \mathbf{{q}} )}, \,\,\, \mathrm{{where}} \,\,\, \alpha \in {\mathbb {R}} \end{aligned}$$

allows exploring a family of measures, as $\mathcal{{F}} ( \mathbf{{q}} )$ can be designed to constrain integration to those configurations which do not violate triangular inequalities, and moreover can be chosen so as to remove non realistic simplices. The characteristic partition function of the model becomes then

$$\begin{aligned} Z = \left[ \prod _\ell ^\mathcal{{N}} \int\limits _0^\infty {\mathrm{d}} q_\ell \, q_\ell ^{- \alpha } \right] \, \mathcal{{F}} ( \mathbf{{q}} ) \, \mathrm{{e}}^{- \sum _s A_s ( \mathbf{{q}} )} \; , \end{aligned}$$

where $\mathcal{{N}}$ is the number of links and $A_s$ is the contribution to the action of simplex $s$.

It is worth recalling that in (Desale and Ramanan 1975) arithmetic techniques and the Weil conjecture were used, and a crucial ingredient was the property that the volume of a particular locally symmetric space attached to $SL_n$ with respect to the canonical measure—an invariant known as the Tamagawa number of $SL_n$—equals 1. The simplicial volume is a homotopy invariant of oriented, closed, connected manifolds defined in terms of the singular chain complex with real coefficients. Such invariant measures the efficiency of representing the fundamental space class using singular simplices. Since the fundamental class is nothing but a generalized triangulation of the manifold, the simplicial volume can be interpreted as well both as a measure for the complexity of the manifold and as a homotopy invariant approximation of the Riemannian volume. $Z(x)$ provides then the generating function (Poincaré polynomial) of the Betti numbers of $\mathcal {A}$.

The final step is to compare the Betti numbers obtained empirically from the data against such generating function, thus determining [simply through the solution of a system of (non-linear) algebraic equations] the set of non-zero $J_{\ell _1 \dots \ell _k \dots \ell _{n+1}}$. This fully determines which antibody influences which, including ‘many-body’ influences, i.e. when and if it may happen that a given set of (two or more than two) antibodies play a role only when simultaneously active.

A short discussion of Regge calculus, meant to introduce in simple way, accessible also to readers not familiar with the notion of geometry over discrete spaces (simplicial complexes), and some of the notions actually used in the derivation can be found in Battaglia and Rasetti (2003), where some of the preliminary ideas of the scheme are described, successively developed in extended way for present and other applications. As for the work in $\mathbb {Z}_2$ quantum gravity which our generalized model of immune system is isomorphic to, a more articulated and complete set of references is available in Giulini (2007) and Bittner et al. (1999).

## A global topological application of the S[B] paradigm

In this section we introduce the $S[B]$ paradigm for modeling complex adaptive systems and we discuss the IS metaphor as a global topological application of the adaptation phase; the aim is to contribute to understand the adaptability feature that, as addressed in the paper of Stepney et al. (2005), still remains ‘poorly understood’.

In the $S[B]$ paradigm a complex system consists of two components, the computation level from where its behavior $B$ emerges, the interactive machine, and the context of the computation, its global structure $S$. Both levels are distinct but entangled in a unique computational model that evolves by learning and adapting. The computational model associated to the $S[B]$ plays a crucial role in the characterization of the adaption phase, it can be represented by any mathematical model of computation, provided that it allows to express the dependency between different levels of abstraction.

**Fig. 2:**
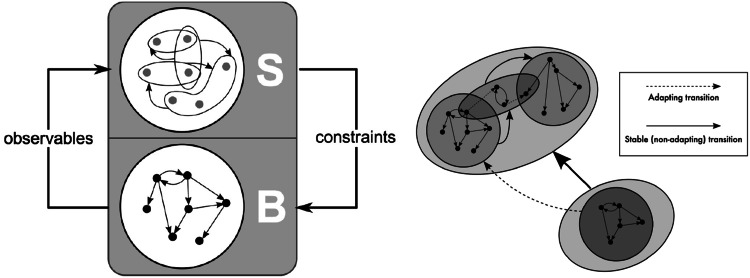
$$S[B]$$ model

Figure 2 shows a simple adaptive system represented by finite state machines, which is the most general among other models, such as complex automata, higher dimensional automata, hypernetworks, recurrent neural network, multiagent, etc. On the left hand side, the two components are entangled in such a way that the emergent behaviour $B$ is subject to the global constraints while the global structure $S$ is affected by the emergent behavior. On the right, an $S[B]$ system is depicted as a light oval $S$ that embeds a dark round $B$, showing the adaptation phase that takes place whenever the computation can no longer evolve in the current context (the $S[B]$ on the lower right corner). The adaptation phase allows $S$ to relax the set of constraints so as to permit further computations—in the figure the black arrow drawn between the two $S$ components, represents the change of the global context, and the dashed arrow between the dark rounds represents the unfolding of the computation. The evolution of such a model relies on the ability of the system to adapt its computation to global requirements.

A full yet concise description of the formal definition of $S[B]$ on a finite state machine that encapsulates both the computation $(B)$ and its controller $(S)$ follows. In this framework, both $B$ and $S$ are classically described as a finite state machine of the form $B=(Q, q_0, \rightarrow _B)$ ($Q$ set of $B$ states, $q_0$ initial $B$ state and $\rightarrow _B$ transition relation) and $S = (R, r_0, \mathcal {O}, \rightarrow _S, L)$ where $R$ is a set of $S$ states, $r_0$ is the initial $S$ state, $\mathcal {O}$ is an observation function of $B$ states, $\rightarrow _S$ is a transition relation and $L$ is a state labeling function. The function $L$ labels each $S$ state with a formula representing a set of constraints over an *observation* of the $B$ states. Therefore, a $S$ state $r$ can be directly mapped to the set of $B$ states satisfying $L(r)$. Through this hierarchy, $S$ can be viewed as a *second-order* structure $(R \subseteq 2^Q, r_0,\rightarrow _S \subseteq 2^Q \times 2^Q, L)$ where each $S$ state $r$ is identified with its corresponding set of $B$ states. An $S[B]$ *system* is the combination of an interactive machine $B=(Q, q_0, \rightarrow _B)$ and a coordinator $S=(R, r_0,\mathcal {O}, \rightarrow _S, L)$ such that for all $q \in Q$, $\mathcal {O}(q) \ne \perp$. In any $S[B]$ system the initial $B$ state must satisfy the constraints of the initial $S$ state, i.e. $q_0 \models L(r_0)$.

During adaptation phase the $B$ machine is no longer regulated by the $S$ controller, except for a condition, called *transition invariant*, that must be fulfilled by the system undergoing adaptation. The complete and formal definition of the $S[B]$ based on finite state machine, its semantics and the *adaptability checking* can be found in Merelli et al. (2013).

**Fig. 3:**
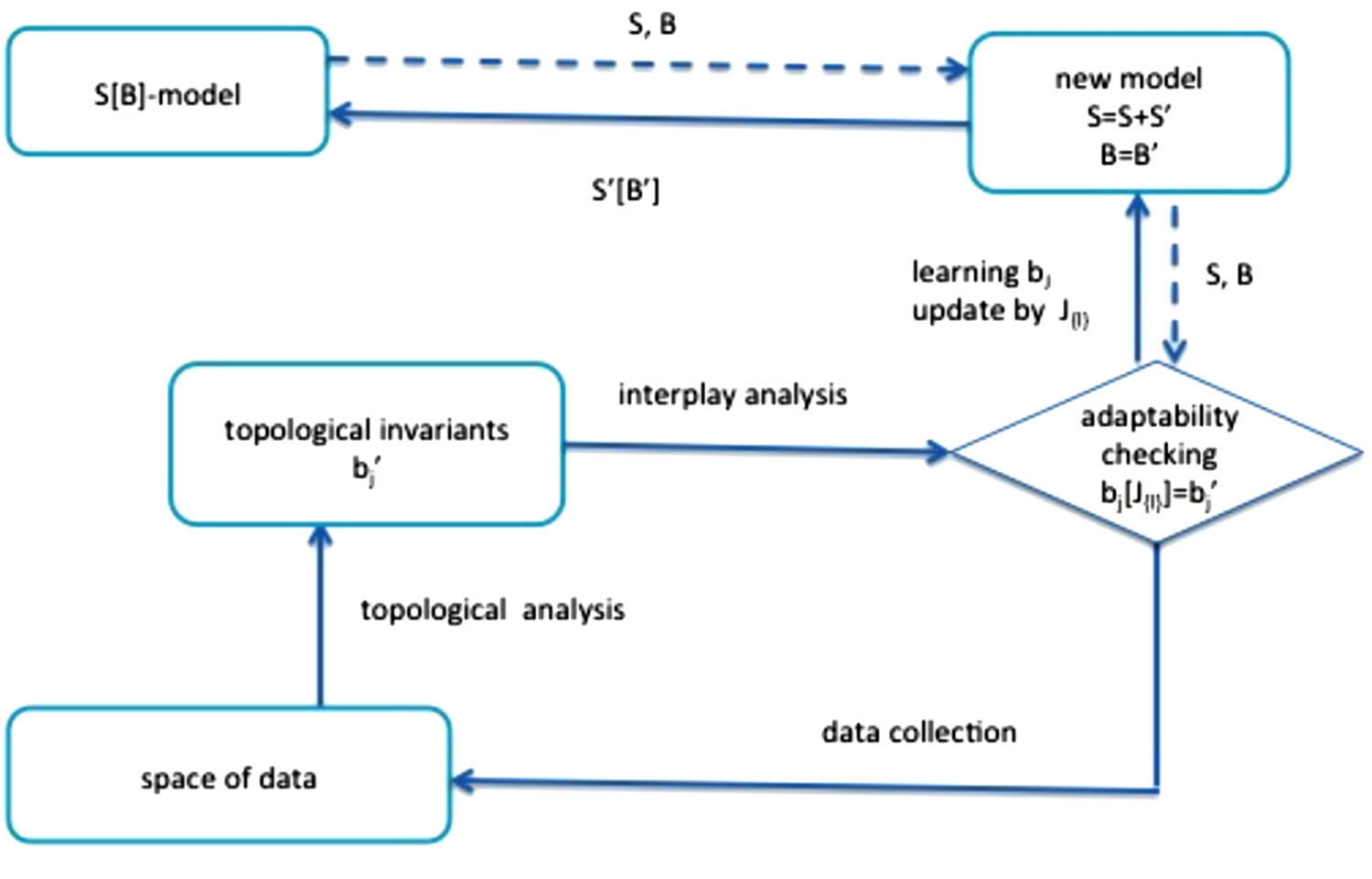
S[B] adaptability checking

**Fig. 5:**
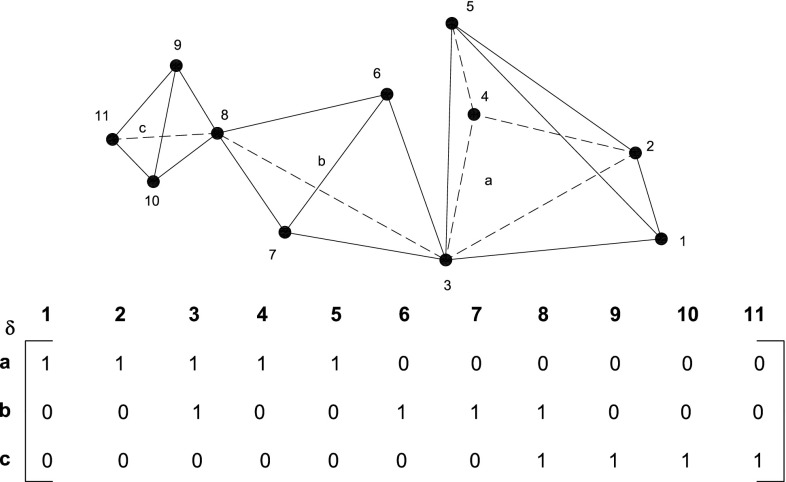
Simplicial complex $\mathcal {C}$ from the graph $\mathcal {G}$

It is quite evident that the model described above can be applied when the system requirements are known a priori and the adaptation phase reduces to dynamic selection of possible states with respect to environmental changes. To overcome this limit and allow the definition of a model that can change the set of global constraints and consequently the set of computations at run-time, we adopt the IS metaphor to characterize the adaptation phase of an $S[B]$ model. The global context is defined as a function of the topological invariants extracted from the analysis of the space of data: the Betti numbers. In the model proposed in previous section the Betti numbers and the $J_{\{\ell \}}$ interaction matrix faithfully represent the relations hidden in the current space of data. Thus, the adaptation phase of an $S[B]$ system is indeed represented as the interplay capabilities of the immune system to identify, classify and learn the new relationship emerging among the actors of the system. Figure 3 graphically mimics the adaptability checking performed by an $S[B]$ system; it starts on the upper left corner of the figure with the actual model $S[B]$ that, when necessary, may be adapted to a new context provided by the topological analysis of the space of data (set of observations of real system). The changes in the context is determined by comparing the Betti numbers of the space of data with the Betti numbers of the actual model. If there is no new knowledge, the model remains $S[B]$ otherwise it adapts to the new context by learning the knowledge provided by the Betti numbers, updating its computation with new set of relations $J_{\{\ell \}}$ and becoming $S'[B']$. This learning process reminds us of what in literature is called *recurrent neural network*, a process based on active exploration of an unknown environment and the generation of a finite state automata model of the environment.

Summarizing, inspired by the IS metaphor we present a computational model as an higher order relational model which deals with multilinear n-body interactions, the interactions characteristic of the immune response. In such case, the model adapts when it no longer fits the space of observed data, and the construction of the topological field model allows us to determine the values of the $J_{{\{\ell \}}}$ matrix, hence, e.g., the classes of antibodies that are in relation in the current immune response. We call this step a recursive construction of a relational model that learns new antibody relations as immune response to the presence of an antigene.

As future work, we aim to apply the proposed approach to real-world IS phenomena treated bot in *silico* and in *vivo* experiments and compare the results with other similar models.

## Concluding remarks

We have defined a new topology-based method suitable to provide a benchmarking application of the S[B] paradigm. The method relies on a multi-linear model of immune system inspired by the topology of space of data. Starting from the notion of an Ising model in a mean field, given by Parisi and others in their seminal work, we proposed a more sophisticated version that is multilinear in the configurational variables (the antibody concentrations) instead of constant or at most linear. This work is not intended to be the study of the dynamics of the immune network in view of establishing the equilibrium among antibodies, but, instead, it has a prospective interest and strategic aim at defining a new approach for the analysis of the immune system as a metaphor of a real-life system represented in terms of Big Data.

## Figures and Tables

**Fig. 4:**
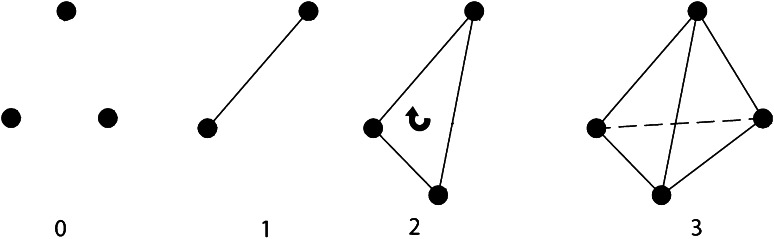
Simplices in $\mathbb {R}^3$. 0-simplex is point or vertex, 1-simplex is an edge, 2-simplex is a triangle, and 3-simplex is a thetrahedron

## Appendix

### Persistent homology and Betti numbers

In this appendix we describe a general approach that allows to extract global topological information from a space of data. It is based on three basic steps: (i) The interpretation of the huge collection of $points$ that constitutes the space of data; this is achieved by resorting to a family of simplicial complexes (Fig. 4), parametrized by some suitably chosen ‘proximity parameter’ (Fig. 5). This operation converts the data set into a global topological object. In order to fully exploit the advantages of topology, the choice of such parameter should be metric independent. In our context it measures the expression of a possible $relation$. (ii) The reduction of noise, affecting the data space, as the result of the parametrized persistent homology. (iii) The encoding of the data set persistent homology in the form of a parameterized version of topological invariants, in particular Betti numbers, i.e. the invariant dimensions of the homology groups. These three steps provide an exhaustive knowledge of the global features of the space of data, even though such a space is neither a metric space nor a vector space, as other approaches require (Carlsson 2009).

In order to better comprehend the scheme, it is necessary to recall that homology is a mathematical tool that ‘measures’ the shape of an object (typically a manifold). The result of this measure is an algebraic object, a succession of groups. Informally, these groups encode the number and the type of ‘holes’ in the manifold. A basic set of invariants of a topological space $X$ is just its collection of homology groups, $H_i(X)$. Computing such groups is certainly non-trivial, even though efficient algorithmic techniques are known to do it systematically. Important ingredients of these techniques, and outcomes as well of the computation, are just Betti numbers; the $i$-th Betti number, $b_i=b_i(X)$, denotes the rank of $H_i(X)$. It is worth remarking that Betti numbers often have an intuitive meaning, for example, $b_0$ is simply the number of connected components of the space considered. While oriented 2-dimensional manifolds are completely classified by $b_1=2g$, where $g$ is the genus (i.e. number of ‘holes’) of the manifold; $b_j$ with $j \ge 2$ classifies the features (number of higher-dimensional *holes*) of higher-dimensional manifolds. What makes them convenient is the fact that in several cases knowing the Betti numbers is the same as knowing the full space homology. Sometimes to know the homology groups it is sufficient to know the corresponding Betti numbers, typically much simpler to compute. In the absence of torsion, if we want to distinguish two topological objects via their homology, their Betti numbers may already do it.

Data can be represented as a collection, unordered sequence, of points in a $n$-dimensional space $E_n$, the space of data. The conventional way to convert a collection of points within a space such as $E_n$ into a global object, is to use the point cloud as the vertex set of a combinatorial graph, $\mathcal {G}$. The edges of the graph are exclusively determined by a given notion of *proximity*, specified by some weight parameter $\delta$. The parameter $\delta$ should not fix a ‘distance’, that would imply fixing some sort of metric, but rather provide information about ‘dependence’, i.e. correlation or, even better, relation. If dependence had to do with distance, it should be a non-metric notion, rather a chemical distance or ontological distance just to mention an example. A graph of this sort, while capturing pretty well connectivity data, essentially ignores a wealth of higher order features beyond clustering. Such features can instead be accurately discerned by thinking of the graph as the *scaffold* (1-skeleton) of a different, higher-dimensional, richer (more complex) discrete object, generated by completing the graph $\mathcal {G}$ to a simplicial complex, $\mathcal {C}$. The latter is a piecewise-linear space built from simple linear constituents (simplices) identified combinatorially along their faces. The decisions as how this is done, implies a choice of how to fill in the higher dimensional simplices of the proximity graph. Such choice is not unique, and different options lead to different global representations. Two among the most natural and common ones, equally effective to our purpose, but with different characteristic features, are: (i) the Čech simplicial complex, where $k$-simplices are all unordered $(k+1)$-tuples of points of the space $E_n$, whose closed $\frac{1}{2} \delta$-ball neighborhoods have a non-empty mutual intersection; (ii) the Rips complex, an abstract simplicial complex whose $k$-simplices are the collection of unordered $(k+1)$-tuples of points pairwise within distance $\delta$. The Rips complex is maximal among all simplicial complexes with the given 1-skeleton (the graph), and the combinatorics of the 1-skeleton completely determines the complex. The Rips complex can thus be stored as a graph and reconstructed out of it. For a Čech complex, on the contrary, one needs to store the entire boundary operator, and the construction is more complex; however, this complex contains a larger amount of information about the topological structure of the data space.

Algebraic topology provides a mature set of tools for counting and collating holes and other topological pattern features, both spaces and maps between spaces, for simplicial complexes. It is therefore able to reveal patterns and structures not easily identifiable otherwise. As persistent homology is generated recursively, corresponding to an increasing sequence of values of $\delta$. Complexes grow with $\delta$. This leads us to naturally identifying the chain maps with a sequence of successive inclusions. Persistent homology is nothing but the image of the homomorphism thus induced. The available algorithms for computing persistent homology groups focus typically on this notion of filtered simplicial complex. Most invariants in algebraic topology are quite difficult to compute efficiently. Fortunately, homology is exceptional under this respect because the invariants arise as quotients of finite-dimensional spaces.

Topological information is contained in persistence homology, that can be determined and presented as a sort of parameterized version of the set of Betti numbers. Its role is just that of providing summaries of information over domains of parameter values, so as to better understand relationships among the geometric objects constructed from data. The emerging geometric/topological relationships involve continuous maps between different objects, and therefore become manifestations of functoriality, i.e, imply the notion that invariants can be extended not just to the objects studied, but also to the maps between such objects. Functoriality is central in algebraic topology because the functoriality of homological invariants is what permits one to compute them from local information. We recall the K$\ddot{u}$nneth theorem that allows to consider the Poincaré polynomial of the space $X$ as the generating function of the Betti numbers of $X$.
